# Metabolic syndrome components and diabetes incidence according to the presence or absence of impaired fasting glucose: The Japan Epidemiology Collaboration on Occupational Health Study

**DOI:** 10.1016/j.je.2016.08.015

**Published:** 2017-04-20

**Authors:** Kayo Kurotani, Toshiaki Miyamoto, Takeshi Kochi, Masafumi Eguchi, Teppei Imai, Akiko Nishihara, Kentaro Tomita, Akihiko Uehara, Makoto Yamamoto, Taizo Murakami, Chii Shimizu, Makiko Shimizu, Satsue Nagahama, Tohru Nakagawa, Toru Honda, Shuichiro Yamamoto, Hiroko Okazaki, Naoko Sasaki, Ai Hori, Chihiro Nishiura, Keisuke Kuwahara, Reiko Kuroda, Shamima Akter, Ikuko Kashino, Akiko Nanri, Isamu Kabe, Tetsuya Mizoue, Naoki Kunugita, Seitaro Dohi

**Affiliations:** a National Center for Global Health and Medicine, Tokyo, Japan; b Nippon Steel & Sumitomo Metal Corporation Kimitsu Works, Chiba, Japan; c Furukawa Electric Co., Ltd., Tokyo, Japan; d Azbil Corporation, Tokyo, Japan; e Mitsubishi Plastics, Inc., Tokyo, Japan; f Yamaha Corporation, Shizuoka, Japan; g Mizue Medical Clinic, Keihin Occupational Health Center, Kanagawa, Japan; h All Japan Labour Welfare Foundation, Tokyo, Japan; i Hitachi, Ltd., Ibaraki, Japan; j Mitsui Chemicals, Inc., Tokyo, Japan; k Mitsubishi Fuso Truck and Bus Corporation, Kanagawa, Japan; l Tokyo Gas Co., Ltd., Tokyo, Japan; m Teikyo University, Tokyo, Japan; n The University of Tokyo, Tokyo, Japan; o National Institute of Public Health, Saitama, Japan

**Keywords:** Diabetes, Metabolic syndrome, Impaired fasting glucose, Number of components, Japanese

## Abstract

**Background:**

We prospectively examined the association of diabetes risk with the number of metabolic abnormalities, as well as their combinations, according to the presence or absence of impaired fasting glucose (IFG) in a large-scale Japanese working population.

**Methods:**

Participants included 55,271 workers at 11 companies who received periodic health check-ups between 2008 and 2013. The metabolic syndrome (MetS) components were defined using the 2009 Joint Interim Statement. IFG was defined as fasting plasma glucose 5.6–6.9 mmol/L. Diabetes newly diagnosed after the baseline examination was defined according to the American Diabetes Association criteria. We calculated the hazard ratios (HRs) for diabetes incidence using the Cox proportional hazards model.

**Results:**

During the follow-up period (median 4.95 years), 3183 subjects developed diabetes. In individuals with normal fasting glucose levels, the risk of diabetes increased steadily with the increasing number of MetS components; the multivariable-adjusted HRs for incident diabetes for the number of MetS components were 2.0, 4.3, 7.0, and 10.0 for one, two, three, or four MetS components, respectively, compared with the absence of components. A similar association was observed among individuals with IFG; the corresponding HRs were 17.6, 23.8, 33.9, and 40.7. The combinations that included central obesity appeared to be more strongly associated with diabetes risk than other combinations with the same number of MetS components within the same glucose status.

**Conclusions:**

Our findings indicate that risk stratification of individuals by the presence or absence of IFG and the number of MetS components can detect individuals with a high risk of diabetes.

## Highlights

• We examined diabetes risk by the number and combination of metabolic abnormalities.
• Diabetes risk increased with the number of abnormalities for a given glucose status.
• The combinations that included central obesity were strongly associated with diabetes.

## Introduction

Metabolic syndrome (MetS) is a cluster of risk factors, including raised blood pressure, dyslipidemia, impaired fasting glucose (IFG), and central obesity.^1^, ^2^ Diabetes risk has been shown to increase with the number of MetS components.^3^, ^4^, ^5^, ^6^ Given that IFG is an early stage of developing diabetes^7^ and a strong predictor of diabetes,^8^ it would be preferable to treat the glucose component separately from other components in stratifying diabetes risk. Few studies, however, have adopted this analytic strategy in assessing diabetes risk in relation to the number of metabolic abnormalities.^9^ In addition to the number of MetS components, it remains unclear whether specific combinations of the components confer higher risks of diabetes. Wilson et al reported that combinations that included central obesity and IFG were more strongly associated with diabetes risk than others with the same number of components.^5^, ^10^ However, that study was relatively small in size (n ≈ 2500), so a larger study is required to provide a more stable risk estimate for each combination of metabolic components. Here, we prospectively examined the association of diabetes risk with the number of MetS components, as well as their combinations, according to the presence or absence of IFG in a large-scale multi-center cohort of Japanese male and female workers.

## Materials and methods

### Study procedure

The Japan Epidemiology Collaboration on Occupational Health (J-ECOH) Study is an ongoing multicenter epidemiologic study among workers of 12 companies in Japan.^11^, ^12^ In Japan, employees are obliged to undergo a general health examination at least once a year under the Industrial Safety and Health Act. As of March 2015, 11 of 12 participating companies provided data from health check-ups that were conducted between January 2008 and December 2013 or between April 2008 and March 2014. The date of the earliest examination (mostly in 2008) was regarded as the baseline, but if the 2008 dataset contained a large amount of missing data, the data of the 2009 or 2010 examination was used as the baseline (for two companies). Subjects were followed from the baseline until the date of the most recent examination (maximally December 2013 or March 2014). The study protocol was approved by the Ethics Committee of the National Center for Global Health and Medicine, Japan. The requirement for written informed consent was waived.

### Subjects

Of the 95,040 subjects with baseline data, we excluded 1532 subjects aged under 20 years, 15,660 subjects with missing data regarding the diagnosis of diabetes, and 5339 subjects with diabetes, which was defined as a fasting plasma glucose level of ≥7.0 mmol/L (126 mg/dL), casual plasma glucose level of ≥11.1 mmol/L (200 mg/dL), hemoglobin A1c (HbA1c) of ≥48 mmol/mol (6.5%), or current use of an anti-diabetic drug, in accordance with the American Diabetes Association criteria.^13^ After further exclusion of 13,695 subjects with missing data on waist circumference, fasting triglyceride, high density lipoprotein (HDL)-cholesterol, blood pressure, current use of cholesterol-lowering drugs and anti-hypertensive drugs, or smoking status, 58,814 subjects remained. We further excluded 3543 subjects who did not attend any subsequent health check-ups, leaving 55,271 subjects (47,160 men and 8111 women) for the analysis. Compared with those who were included in the present study, those who were excluded (n = 3543) were older and had higher levels of blood pressure and triglyceride and higher proportions of antihypertensive and hypolipidemic drug use.

### Assessment of the MetS components

The MetS components were defined according to the 2009 Joint Interim Statement^1^, ^14^, ^15^: 1) waist circumference ≥90 cm in men and ≥80 cm in women (for Asians, including Japanese), 2) triglyceride level ≥1.7 mmol/L (150 mg/dL) or current medication for dyslipidemia, 3) HDL-cholesterol level <1.04 mmol/L (40 mg/dL) in men and <1.3 mmol/L (50 mg/dL) in women, 4) blood pressure ≥130 mm Hg systolic or ≥85 mm Hg diastolic or current use of anti-hypertensive drugs, and 5) fasting plasma glucose level ≥5.6 mmol/L (100 mg/dL). Additionally, IFG was defined as fasting plasma glucose 5.6–6.9 mmol/L (100–125 mg/dL).^16^

### Laboratory measurements

In the participating companies, HbA1c was measured using a latex agglutination immunoassay, the HPLC method, or the enzymatic method. Plasma glucose was measured using the enzymatic method or the glucose oxidase peroxidative electrode method, and HDL-cholesterol and triglycerides were measured using the enzymatic method. All laboratories involved in the health checkup at the participating companies have received satisfactory results (rank A or score >95 out of 100) from external quality control surveillance.

### Statistical analysis

We calculated the person-time from the date of the baseline examination to the first date when diabetes was confirmed at a follow-up examination or to the date of the most recent examination, whichever came first. We also calculated the hazard ratios (HRs) and 95% confidence intervals (CIs) for the incidence of diabetes using Cox proportional-hazards regression models by individual MetS component and by the number of MetS components according to the presence or absence of IFG. In the multivariable-adjusted model, we adjusted for age (years, continuous), sex, company, and smoking status (non-smoker or current smoker). We also calculated the HRs for each combination of MetS components. In that analysis, we created a dyslipidemia component if the subject had high triglyceride and/or low HDL-cholesterol level, in accordance with the definition of dyslipidemia in the 2009 Joint Interim Statement.^1^ A two sided P < 0.05 was considered statistically significant. The SAS software package (version 9.3; SAS Institute, Cary, NC, USA) was used to perform all statistical analyses.

## Results

At baseline, the mean (standard deviation) age was 45.5 (9.0) years and 43.8 (9.2) years in men and women, respectively (Table 1). During the follow-up period (median follow-up period: 4.95 years), 3183 subjects developed diabetes. The mean (standard deviation) age at onset of diabetes was 51.8 (7.6) years. Of the MetS components, IFG was the most strongly associated with diabetes; the multivariable-adjusted HRs of incident diabetes were 8.8 (95% CI, 9.0–9.6), 2.5 (95% CI, 2.4–2.7), 2.0 (95% CI, 1.8–2.1), 1.9 (95% CI, 1.8–2.1), and 1.9 (95% CI, 1.7–2.1) for IFG, central obesity, high blood pressure, high triglycerides, and low HDL-cholesterol, respectively.

**Table 1:** Baseline characteristics of subjects.

	Men	Women
n	47,160	8111
Age, years^a^	45.5 (9.0)	43.8 (9.2)
Waist circumference, cm^a^	83.2 (8.4)	75.6 (9.1)
Systolic blood pressure, mm Hg^a^	121.8 (15.0)	115.6 (16.1)
Diastolic blood pressure, mm Hg^a^	77.3 (10.5)	71.7 (10.7)
Antihypertensive drug use, %	9.8	4.5
Fasting glucose, mg/dL^b^	96.0 (91.0–102.0)	90.0 (86.0–96.0)
Triglyceride, mg/dL^b^	104.0 (73.0–153.0)	65.0 (49.0–89.0)
HDL-cholesterol, mg/dL^a^	56.9 (14.4)	69.5 (15.5)
Hypolipidemic medication, %	4.7	3.3
Current smoker, %	40.8	10.8

a Mean (standard deviation).

b Median (interquartile range).

**Fig. 1:**
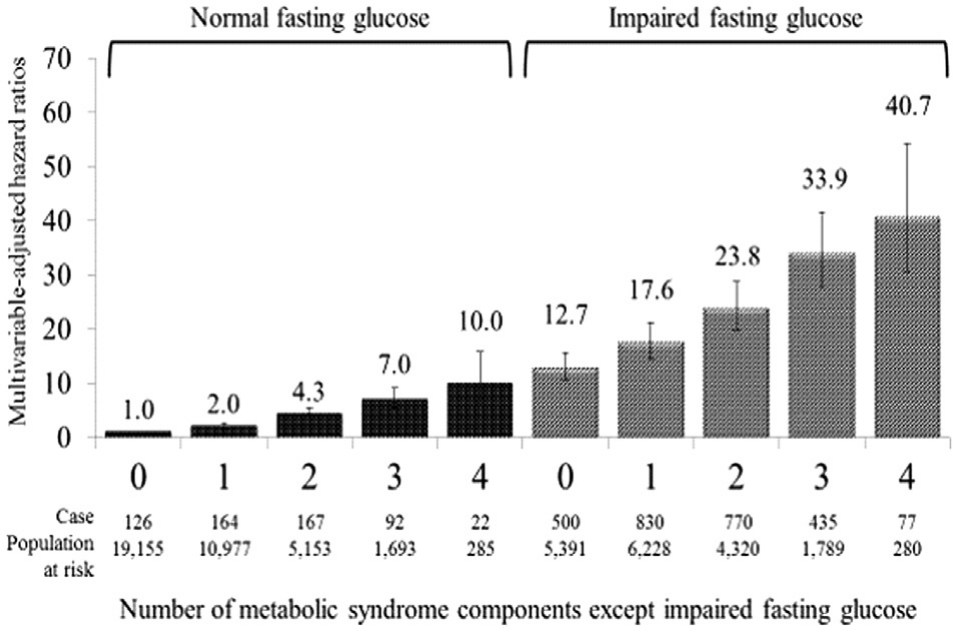
Multivariable-adjusted hazard ratios for the development of diabetes associated with the number of metabolic syndrome components according to the presence or absence of impaired fasting glucose. Adjusted for age, sex, company, and smoking status.

As shown in Fig. 1, in individuals with normal fasting plasma glucose levels, the risk of diabetes increased steadily with the increasing number of MetS components; the multivariable-adjusted HRs for incident diabetes of the number of MetS components from 1 to 4 were 2.0 (95% CI, 1.6–2.6), 4.3 (95% CI, 3.4–5.4), 7.0 (95% CI, 5.3–9.2), and 10.0 (95% CI, 6.4–15.8), respectively, compared with the absence of components. A similar association was observed among individuals with IFG; the multivariable-adjusted HRs for diabetes of the number of metabolic components from 1 to 4 were 17.6 (95% CI, 14.5–21.2), 23.8 (95% CI, 19.6–28.8), 33.9 (95% CI, 27.8–41.5), and 40.7 (95% CI, 30.6–54.1), respectively, compared with the absence of the component. Individuals with IFG alone (HR 12.7; 95% CI, 2.28–2.77) had a higher risk of diabetes than those with all other MetS components.

Table 2 shows the HRs of diabetes for each combination of MetS components, including IFG, central obesity, high blood pressure, and dyslipidemia. Among individuals without IFG, the presence of one of the other MetS components (central obesity, high blood pressure, or dyslipidemia) was associated with a 2-fold higher risk of diabetes compared with the absence of the components. Among normal-glucose individuals who had two MetS components, the combinations that included central obesity had a 68–74% higher risk of diabetes than those without central obesity (i.e., those with high blood pressure and dyslipidemia only). Among individuals with two metabolic abnormalities in addition to IFG, the combinations that included central obesity were associated with a 23–25% higher risk of diabetes than those without central obesity (i.e., the combination that included both high blood pressure and dyslipidemia). The combinations that included central obesity appear to be more strongly associated with diabetes risk than others with the same number of MetS components within the same glucose status.

**Table 2:** Multivariable-adjusted hazard ratios for the development of diabetes associated with the combination of metabolic syndrome components.

Number of components, except impaired fasting glucose	Impaired fasting glucose	Central obesity	High blood pressure	Dyslipidemia^a^	Population at risk (n)	Number of events	Multivariable-adjusted hazard ratio (95% CI)^b^
Without impaired fasting glucose							
0	(−)	(−)	(−)	(−)	19,155	126	Reference
1	(−)	(+)	(−)	(−)	2210	33	2.2 (1.5–3.3)
1	(−)	(−)	(+)	(−)	5142	71	1.8 (1.4–2.5)
1	(−)	(−)	(−)	(+)	4240	70	2.2 (1.6–2.9)
2	(−)	(+)	(+)	(−)	1527	62	5.7 (4.2–7.7)
2	(−)	(+)	(−)	(+)	1586	68	5.9 (4.4–7.9)
2	(−)	(−)	(+)	(+)	2049	57	3.4 (2.5–4.7)
3	(−)	(+)	(+)	(+)	1354	84	8.0 (6.0–10.5)
With impaired fasting glucose							
0	(+)	(−)	(−)	(−)	5391	500	12.6 (10.4–15.4)
1	(+)	(+)	(−)	(−)	1045	145	18.9 (14.9–24.1)
1	(+)	(−)	(+)	(−)	3282	442	17.4 (14.2–21.3)
1	(+)	(−)	(−)	(+)	2243	312	18.1 (14.7–22.4)
2	(+)	(+)	(+)	(−)	1423	277	26.6 (21.5–32.9)
2	(+)	(+)	(−)	(+)	1086	208	26.0 (20.8–32.5)
2	(+)	(−)	(+)	(+)	1909	317	21.2 (17.1–26.1)
3	(+)	(+)	(+)	(+)	1629	411	35.4 (28.9–43.3)

a Dyslipidemia included triglyceride level ≥1.7 mmol/L (150 mg/dL), HDL-cholesterol level <1.04 mmol/L (40 mg/dL) in men and <1.3 mmol/L (50 mg/dL) in women, or current medication for dyslipidemia.

b Adjusted for age, sex, company, and smoking status.

## Discussion

In a large-scale cohort of the working population in Japan, we found that the risk of diabetes incidence sharply increased with the increasing number of MetS components and that the combinations that included the central obesity component were more strongly associated with diabetes risk than others with the same number of metabolic abnormalities with the same glucose status. This study is among few prospective investigations of diabetic risk in relation to specific combinations of MetS components.

Our findings of a steadily increasing trend of diabetes risk with the number of MetS components for both IFG and non-IFG are in line with those of Japanese population-based studies.^9^, ^10^ As expected, those with IFG had a much higher risk of diabetes than those without IFG for a given number of MetS components; the diabetes risk was 6.3-, 4.1-, 3.4-, and 3.4-fold higher in those with IFG than that in those without IFG for individuals with 1–4 metabolic abnormalities, respectively. In the Framingham Offspring Study, Wilson et al showed that the combinations of MetS components that included IFG were associated with an approximately 2-fold higher risk of diabetes than other combinations with the same number of MetS components.^5^ Although it is not feasible to directly compare the relative risk between that study and ours due to the different definition of the comparison group, the results of both studies support the importance of considering the glucose level separately from other MetS components in stratifying diabetes risk.

In the present study, the combinations that included central obesity were associated with a 23–74% higher risk of diabetes than others with the same number of MetS components within the same glucose status, which is consistent with the results of Wilson's study,^5^ in which the corresponding value was 17–61%. Similarly, the Niigata Wellness study in Japan showed that the combinations that included central obesity were associated with a 41–134% higher risk of diabetes than others with the same number of MetS components.^10^ These findings suggest that there might be a combined effect of central obesity and other metabolic abnormalities on the development of diabetes among those with the same number of MetS components within the same glucose status. Taken together with our overall results, as shown in Fig. 1 and Table 2, diabetes risk was largely determined via IFG and the number of MetS components, and the difference in diabetes risk was small among groups that had the same fasting glucose status and the same number of MetS components.

The major strengths of the present study included the enrollment of a large number of participants from several Japanese companies. The limitations of the present study should also be mentioned. First, the follow-up period was relatively short. The incidence rate and relative risk in the present study, however, are comparable to those in a long-term prospective study in Japan.^9^, ^10^ Second, the definition of MetS components was based on a single measurement at baseline. Because specific health checkups and specific counseling focusing on MetS have been conducted since 2008 in Japan,^17^ individuals with MetS at baseline might have been instructed to modify their lifestyle. If so, risk estimates would be attenuated towards null. Third, potential confounding variables, including dietary intake and physical activity, were not assessed in this study. Finally, most subjects in the present study were middle-aged employees of large companies, so our findings may not be generalized to employees of small-to medium-sized companies, the self-employed, and the unemployed. Give that age-specific prevalence of diabetes in the present study population was similar to that in a nationally-representative sample,^12^, ^18^ however, we speculate that the association observed in the present study may be replicated in the general population.

In summary, the present study indicates that considering the presence or absence of IFG and the number of MetS components can detect individuals with a high risk of future diabetes. Among individuals with the same number of MetS components within the same glucose status, the combinations that included central obesity might identify a high-risk population of diabetes.

## Sources of support

This study was supported by the Industrial Health Foundation, JSPS KAKENHI Grant Number 25293146, 16H05251, and Industrial Disease Clinical Research Grants (140202-01), and the Grant of National Center for Global Health and Medicine (28-Shi-1206).

## Conflicts of interest

None declared.

## References

[1] Alberti K.G., Eckel R.H., Grundy S.M. (2009). Harmonizing the metabolic syndrome: a joint interim statement of the International Diabetes Federation Task Force on Epidemiology and Prevention; National Heart, Lung, and Blood Institute; American Heart Association; World Heart Federation; International Atherosclerosis Society; and International Association for the Study of Obesity. Circulation.

[2] National Cholesterol Education Program (NCEP) Expert Panel on Detection, Evaluation, and Treatment of High Blood Cholesterol in Adults (Adult Treatment Panel III) (2002). Third Report of the National Cholesterol Education Program (NCEP) Expert Panel on Detection, Evaluation, and Treatment of High Blood Cholesterol in Adults (Adult Treatment Panel III) final report. Circulation.

[3] Nakanishi N., Takatorige T., Fukuda H. (2004). Components of the metabolic syndrome as predictors of cardiovascular disease and type 2 diabetes in middle-aged Japanese men. Diabetes Res Clin Pract.

[4] Sattar N., Gaw A., Scherbakova O. (2003). Metabolic syndrome with and without C-reactive protein as a predictor of coronary heart disease and diabetes in the West of Scotland Coronary Prevention Study. Circulation.

[5] Wilson P.W., D'Agostino R.B., Parise H. (2005). Metabolic syndrome as a precursor of cardiovascular disease and type 2 diabetes mellitus. Circulation.

[6] Wannamethee S.G., Shaper A.G., Lennon L. (2005). Metabolic syndrome vs Framingham Risk Score for prediction of coronary heart disease, stroke, and type 2 diabetes mellitus. Archives Intern Med.

[7] Heianza Y., Arase Y., Fujihara K. (2012). Screening for pre-diabetes to predict future diabetes using various cut-off points for HbA(1c) and impaired fasting glucose: the Toranomon Hospital Health Management Center Study 4 (TOPICS 4). Diabet Med.

[8] Levitzky Y.S., Pencina M.J., D'Agostino R.B. (2008). Impact of impaired fasting glucose on cardiovascular disease: the Framingham Heart Study. J Am Coll Cardiol.

[9] Mukai N., Doi Y., Ninomiya T. (2009). Impact of metabolic syndrome compared with impaired fasting glucose on the development of type 2 diabetes in a general Japanese population: the Hisayama study. Diabetes Care.

[10] Heianza Y., Kato K., Kodama S. (2015). Risk of the development of Type 2 diabetes in relation to overall obesity, abdominal obesity and the clustering of metabolic abnormalities in Japanese individuals: does metabolically healthy overweight really exist? The Niigata Wellness Study. Diabet Med.

[11] Hori A., Nanri A., Sakamoto N. (2014). Comparison of body mass index, waist circumference, and waist-to-height ratio for predicting the clustering of cardiometabolic risk factors by age in Japanese workers–Japan Epidemiology Collaboration on Occupational Health study. Circulation J.

[12] Uehara A., Kurotani K., Kochi T. (2014). Prevalence of diabetes and pre-diabetes among workers: Japan Epidemiology Collaboration on Occupational Health study. Diabetes Res Clin Pract.

[13] American Diabetes Association (2010). Diagnosis and classification of diabetes mellitus. Diabetes Care.

[14] Hu H., Kurotani K., Sasaki N. (2016). Optimal waist circumference cut-off points and ability of different metabolic syndrome criteria for predicting diabetes in Japanese men and women: Japan Epidemiology Collaboration on Occupational Health Study. BMC Public Health.

[15] Kaneko M., Suzuki H., Watanabe H. (2012). Metabolic syndrome is a poor predictor of incident Diabetes Compared with Hemoglobin A1c (Hba1c) in a general Japanese population. J Diabetes Metabolism.

[16] The expert committee on the diagnosis and classification of diabetes mellitus (2003). Report of the expert committee on the diagnosis and classification of diabetes mellitus. Diabetes Care.

[17] Ministry of Health, Labour, and Welfare, Japan (2013). Specific Health Checkups and Specific Health Guidance. Secondary Specific Health Checkups and Specific Health Guidance. http://www.mhlw.go.jp/english/wp/wp-hw3/dl/2-007.pdf.

[18] Kenko Eiyo Joho Kenkyukai (2010). The National Health and Nutrition Survey in Japan, 2007.

